# Bacterial EPIYA effectors – Where do they come from? What are they? Where are they going?

**DOI:** 10.1111/cmi.12040

**Published:** 2012-11-01

**Authors:** Takeru Hayashi, Hiroko Morohashi, Masanori Hatakeyama

**Affiliations:** Division of Microbiology, Graduate School of Medicine, The University of Tokyo7-3-1 Hongo, Bunkyo-ku, Tokyo, 113-0033, Japan

## Abstract

Recent studies have revealed a distinct class of bacterial effectors defined by the presence of EPIYA or EPIYA-related motif. These bacterial EPIYA effectors are delivered into host cells via type III or IV secretion, where they undergo tyrosine phosphorylation at the EPIYA motif and thereby manipulate host signalling by promiscuously interacting with multiple SH2 domain-containing proteins. Up to now, nine EPIYA effectors have been identified from various bacteria. These effectors do not share sequence homology outside the EPIYA motif, arguing against the idea that they have common ancestors. A search of mammalian proteomes revealed the presence of a mammalian EPIYA-containing protein, Pragmin, which potentiates Src family kinase (SFK) activity by binding and sequestrating the SFK inhibitor Csk upon EPIYA phosphorylation. As several bacterial EPIYA effectors also target Csk, they may have evolved through generation of sequences that mimic the Pragmin EPIYA motif. EPIYA motifs are often diverged through multiple duplications in each bacterial effector. Such a structural plasticity appears to be due to intrinsic disorder of the EPIYA-containing region, which enables the bacterial effectors to undergo efficient phosphorylation and mediate promiscuous interaction with multiple host proteins. Given the functional versatility of the EPIYA motif, many more bacterial EPIYA effectors will soon be emerging.

## Introduction

Many bacteria can manipulate their environment by the secretion of proteins (bacterial effectors), which are delivered outside the bacterial cells. Seven different secretion systems (types I–VII) have so far been described. Type III and IV systems allow penetration of the plasma membrane and delivery of bacterial molecules directly into the cytoplasm of target cells. The type III secretion system (TTSS) utilizes a flagellum-like tube to translocalize effector proteins into eukaryotic host cells, whereas the type IV secretion system (TFSS) employs a pilus-based structure to mediate delivery of DNA or proteins into target cells. Once delivered, bacterial effectors elicit pathogenic actions by manipulating host cell signalling. Recent studies have revealed a distinct class of bacterial effectors that undergo tyrosine phosphorylation upon delivery into the eukaryotic host cells, where they interact with a variety of host SH2 domain-containing proteins in a tyrosine phosphorylation-dependent manner. Of special interest is that the tyrosine phosphorylation sites of these bacterial effectors are characterized by the presence of the Glu-Pro-Ile-Tyr-Ala (EPIYA) sequence (EPIYA motif) or sequences closely related to the EPIYA motif, in which the tyrosine residue serves as a phosphorylation site (Backert and Selbach, 2005). Surprisingly, these bacterial EPIYA effectors do not share sequence homology among each other outside the EPIYA motif, indicating they have arisen from convergent evolution, not common descent. In this review, we describe recent advances in our understanding of this new class of bacterial effectors that provide insights into their possible evolutionary origins, structural basis for their functions, and future expansion of this family in both members and functions.

## Bacterial EPIYA effectors

Since the discovery of the archetypal EPIYA effector, *Helicobacter pylori* CagA, research has progressed rapidly to identify bacterial EPIYA effectors as they play crucial roles in disease manifestations during pathogenic bacterial infection. For instance, infection with *H. pylori* strains producing CagA is the strongest risk factor for the development of gastric adenocarcinoma. The EPIYA effector of enteropathogenic *Escherichia coli* (EPEC), Tir, enables invasion of the bacteria into non-phagocytic epithelial cells (Rosenshine *et al*., 1992), and the *Haemophilus ducreyi* EPIYA effector LspA was shown to be required for the development of chancroid in a rabbit infection model (Ward *et al*., #b^9001^). Neutralization of the *Anaplasma phagocytophilum* EPIYA effector, AnkA, using an anti-AnkA antibody abolishes the ability of *A. phagocytophilum* to infect host cells (Lin *et al*., 2007), indicating that bacterial EPIYA effectors are potential therapeutic targets. Nine bacterial EPIYA effectors have so far been detected.

### *Helicobacter pylori* CagA

**Fig. 1:**
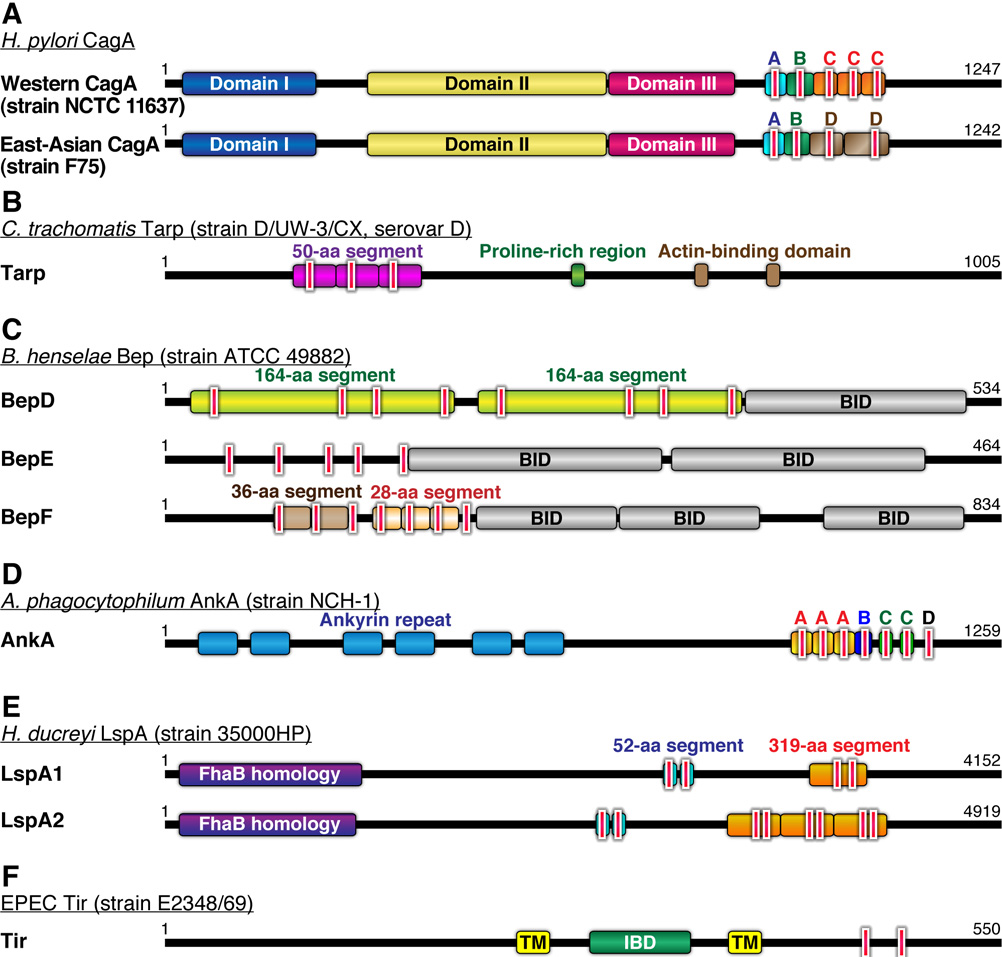
Schematic views of bacterial EPIYA effectors. EPIYA or EPIYA-related motifs are present in multiple numbers in bacterial EPIYA effectors. Each EPIYA-related motif is highlighted with a red box fringed with white. A. *Helicobacter pylori* CagA derived from strain NCTC 11637 (Western CagA) and strain F75 (East Asian CagA). The C-terminal region of Western CagA comprises EPIYA-A segment, EPIYA-B segment and multiple copies (usually one to four copies) of the 34-amino-acid EPIYA-C segments in the C-terminal region. On the other hand, the C-terminal region of East Asian CagA consists of EPIYA-A, -B and -D segments in this order. A very few East Asian CagA species contain multiple numbers of the EPIYA-D segment. Domains I–III in the N-terminal region are defined by the CagA crystal structure. B. *Chlamydia trachomatis* Tarp derived from strain D/UW-3/CX (serovar D). Tarp contains EPIYA-related ENIYE motifs, each of which is embedded in the conserved ∼50-amino-acid segment. The segment is variably duplicated (one to nine times) in the N-terminal region among distinct Tarp serovars. Proline-rich region and WAVE2-like actin-binding domain are also indicated. C. *Bartonella henselae* BepD, BepE and BepF derived from strain ATCC 49882. BepD contains two 164-amino-acid segments, each of which has one EPLYA motif, two NPLYE motifs and one EHLYA motif. BepE contains several EPIYA-related motifs in the N-terminal region. BepF contains 36-amino-acid segments and 28-amino-acid segments, each of which has a single EPIYA-related TPLYA or EPLYA motif. Bep intracellular delivery (BID) domains, which are located in the C-terminal region of each Bep protein, are also indicated. D. *Anaplasma phagocytophilum* AnkA derived from strain NCH-1. AnkA contains multiple repeats of 27-amino-acid EPIYA-A segments, each containing a single ESIYE motif. The EPIYA segments are followed by a 17-amino-acid EPIYA-B segment repeats containing a single EDLYA motif. The repeat numbers of EPIYA-A and -B are variable among distinct AnkA species. In contrast, the two 11-amino-acid EPIYA-C segments and the single EPIYA-D motif are conserved. E. *Haemophilus ducreyi* LspA proteins derived from strain 35000HP. LspA1 and LspA2 contain two EPIYA-related EPIYG motifs, each of which is embedded in the conserved 52-amino-acid segment present in the mid-region. The C-terminal region of LspA1 also possesses a 319-amino-acid segment containing two EPIYA-related EPVYA motifs, which is tandemly duplicated three times in the C-terminal LspA2. FhaB homology domain at the N-terminal region is also indicated. F. Enteropathogenic *E. coli* (EPEC) Tir derived from strain E2348/69. Tir contains EPIYA-related VNPYA and EHIYD motifs in the C-terminal cytoplasmic domain. Extracellular intimin-binding domain (IBD) is present at central region surrounded by two transmembrane (TM) domains.

*Helicobacter pylori* is a spiral-shaped, Gram-negative bacillus colonizing the human stomach. Chronic infection with *H. pylori*, especially those producing the CagA protein, is a primary cause of atrophic gastritis and peptic ulcerations. *H. pylori cagA*-positive strains are also critically involved in the development of gastric carcinoma, the second leading cause of cancer-associated deaths worldwide. CagA is the archetypal bacterial EPIYA effector, which is delivered into the cytoplasm of gastric epithelial cells via the VirB/VirD4 TFSS (Covacci and Rappuoli, 2000). Delivered CagA undergoes sequential tyrosine phosphorylation, initially by Src family kinases (SFKs) and then by c-Abl kinase, at the EPIYA sequence that is present in a variable number in the C-terminal region (Poppe *et al*., 2007; Tammer *et al*., 2007). Based on the sequences flanking each of the EPIYA motifs, four distinct EPIYA segments (EPIYA-A to -D) are defined (Fig. 1A) (Hatakeyama, 2004). The C-terminal region of CagA from *H. pylori* circulating in East Asian counties (Japan, Korea, China) is characterized by the tandem arrangement of EPIYA-A, EPIYA-B and EPIYA-D segments, whereas that of CagA from *H. pylori* circulating in the rest of the world comprises EPIYA-A, EPIYA-B and a variable number (one to four) of EPIYA-C segments in tandem. Upon tyrosine phosphorylation, the EPIYA-C or EPIYA-D segment serves as a specific binding site for the SH2 domain-containing tyrosine phosphatase SHP2, a *bona fide* human oncoprotein (2002a). This CagA–SHP2 interaction deregulates SHP2 phosphatase activity, which in turn elicits aberrant activation of Erk MAP kinase signalling. The EPIYA-D segment binds to SHP2 more strongly than the EPIYA-C segment does (Hatakeyama, 2004). Tyrosine-phosphorylated EPIYA-A or EPIYA-B segment serves as a binding site for the SH2 domain of the C-terminal Src kinase (Csk) (Tsutsumi *et al*., 2003). This CagA–Csk interaction activates Csk, which in turn inhibits SFK activity through tyrosine phosphorylation at the C-terminal inhibitory site. The EPIYA-B segment additionally binds to the p85 regulatory subunit of phosphatidylinositol-3 (PI3) kinase in a tyrosine phosphorylation-dependent manner (Selbach *et al*., 2009). Tyrosine-phosphorylated CagA also interacts with Crk (Suzuki *et al*., 2005), although the EPIYA segment(s) responsible for the Crk interaction is not known. In addition to the above described targets, CagA has also been reported to bind to a large number of proteins in an EPIYA phosphorylation-dependent manner. Those CagA-binding proteins so far reported are listed in Table S1. Notably, however, many of them were reported only once. The list also contains those detected by *in vitro* binding with CagA peptides, which is yet to be confirmed *in vivo*. Nevertheless, incredibly versatile interaction of the bacterial protein with host molecules suggests that, upon delivery, CagA may act as a pathogenic scaffold/adaptor, which simultaneously perturbs multiple host signalling pathways and thereby promotes transformation. Indeed, oncogenic potential of *H. pylori* CagA has been demonstrated by a study using transgenic mice that systemically express CagA. The CagA-transgenic mice spontaneously developed gastrointestinal carcinomas as well as haematological malignancies in a manner that was dependent on EPIYA phosphorylation of CagA (Ohnishi *et al*., 2008). This observation not only provides a formal proof that *H. pylori* CagA is a bacterial oncoprotein but also indicates an important role of CagA–SHP2 interaction, which requires CagA EPIYA phosphorylation, in *in vivo* tumorigenesis. Furthermore, East Asian CagA, which binds to SHP2 more strongly than does Western CagA, was found to be more oncogenic than Western CagA in mice (Miura *et al*., 2009). Thus, variations in the EPIYA segments determine the magnitude of the oncogenic potential of CagA. Also notably, among Western CagA species, those with a larger number of EPIYA-C segments exhibit greater ability to bind to SHP2 and are more frequently associated with gastric carcinoma (Basso *et al*., 2008).

### *Chlamydia trachomatis* Tarp

*Chlamydia trachomatis* is a Gram-negative obligate intracellular bacterium that causes sexually transmitted diseases and blindness. *C. trachomatis* Tarp (translocated actin-recruiting phosphoprotein) is translocated into host epithelial cells by type III secretion. Inside the host cell, Tarp is rapidly tyrosine-phosphorylated at the EPIYA-related ENIYE motif, which is embedded in the conserved ∼50-amino-acid segment that is variably duplicated (one to nine times) in the N-terminal region among distinct serovars (Fig. 1B) (Clifton *et al*., 2004; 2005; Lutter *et al*., 2010). Some of these ENIYE segments comprise double-phosphorylation sites, ENIYENIYE. The Tarp phosphorylation involves multiple host tyrosine kinases such as SFKs, Abl and Syk (Mehlitz *et al*., 2008). Tyrosine-phosphorylated Tarp was originally considered to be involved in cytoskeletal rearrangements of the host cells that lead to endocytosis of the bacteria. However, non-phosphorylated Tarp was still capable of inducing actin polymerization via a WAVE2-like actin-binding domain in the C-terminal region (Clifton *et al*., 2005). Phosphorylation of Tarp on Tyr179, which does not constitute an EPIYA motif, allows interaction with PI3 kinase and VAV2 (Lane *et al*., 2008). Tarp also interacts with Vav2 upon Tyr189 phosphorylation. Currently, roles of these interactions for Tarp action are not known. Additionally, tyrosine-phosphorylated Tarp binds to the SH2 domain-containing adaptor protein SHC1, thereby causing aberrant activation of Erk signalling during entry of *C. trachomatis* into host cells (Mehlitz *et al*., 2010). The Tarp–SHC1 interaction confers resistance to apoptosis to infected epithelial cells via unknown mechanisms. The number of ENIYE repeats appears to be associated with the tissue tropism and disease outcome of *C. trachomatis* infection (Lutter *et al*., 2010). For example, serovar D carrying Tarp^D^, which contains three repeats of the 50-amino-acid segment, mainly infects mucosal epithelial cells. Serovar L2 carrying Tarp^L2^, which contains six such repeats with three containing double ENIYE repeats and three containing only a single repeat of ENIYE, is associated with the invasive sexually transmitted disease lymphogranuloma venereum and infects lymph nodes, causing a more systemic infection. Tarp phosphorylation may be important for host cell invasion as L2 survives longer in macrophages (Yong *et al*., 1987). Molecularly, Tarp^L2^ interacts with SHC1 more strongly than Tarp^D^ does, and Tarp^L2^, but not Tarp^D^, binds to the SH2 domain-containing adaptor protein Nck (Mehlitz *et al*., 2010).

### *Bartonella henselae* BepD, BepE and BepF

*Bartonella henselae* is a Gram-negative, facultative intracellular bacterium that causes human diseases such as cat-scratch disease, bacillary angiomatosis, bacillary peliosis hepatis and neuroretinitis. Bacillary angiomatosis and peliosis are angiomatous lesions characterized by abnormal proliferation of endothelial cells, which occurs almost exclusively in AIDS patients. *B. henselae* injects seven *Bartonella* effector proteins (BepA–BepG) via VirB/VirD4 type IV secretion into vascular endothelial cells (Schulein *et al*., 2005). BepD, BepE and BepF each contain multiple EPIYA-related EPLYA motifs in their N-terminal regions (Fig. 1C) and, indeed, BepD has been shown to be tyrosine-phosphorylated in host endothelial cells. Upon phosphorylation on Tyr37, BepE acquires the ability to interact with Csk. BepE also interacts with SHP2 in a manner that is dependent on Tyr64 phosphorylation (Selbach *et al*., 2009). Although there is evidence that SFK phosphorylates BepE, kinases responsible for Bep phosphorylation *in vivo* remain to be elucidated.

### *Anaplasma phagocytophilum* AnkA

*Anaplasma phagocytophilum* is a Gram-negative intragranulocytic bacterium that causes human granulocytic anaplasmosis (ehrlichiosis), a potentially fatal disease characterized by leucopenia and thrombocytopenia. AnkA, a 160–190 KDa *A. phagocytophilum* protein with ∼11 ankyrin repeats at the N-terminal two-thirds, is delivered into the host cell cytoplasm via the VirB/VirD4 TFSS (Fig. 1D) (Ijdo *et al*., 2007; Lin *et al*., 2007). AnkA contains four different types of EPIYA segments termed EPIYA-A, -B, -C and -D, each of which contains an EPIYA-related motif, at the C-terminal region. Among these, EPIYA-A and EPIYA-B segments vary in number and order, whereas EPIYA-C and EPIYA-D segments are conserved among different strains. These EPIYA motifs are tyrosine-phosphorylated by either SFKs or c-Abl. The tyrosine phosphorylation at the EPIYA motifs allows AnkA to bind to SHP1 (Ijdo *et al*., 2007), an SHP2-related SH2 domain-containing protein tyrosine phosphatase predominantly expressed in haematopoietic cells that is thought to act as a negative regulator in multiple signalling pathways. As is the case of the *H. pylori* CagA–SHP2 interaction, the interaction may induce a conformational change in SHP1 that results in deregulated activation of SHP1 phosphatase activity in infected neutrophils.

### *Haemophilus ducreyi* LspA1 and LspA2

*Haemophilus ducreyi* is a Gram-negative bacillus that causes the sexually transmitted disease chancroid. *H. ducreyi* effector proteins LspA1 (large supernatant protein A1) and LspA2 are known to dampen the phagocytic activity of macrophages and granulocytes (Vakevainen *et al*., 2003). Specifically, these LspA proteins block Fcγ receptor-mediated triggering of phagocytosis by inhibiting SFK activities in host cells (Mock *et al*., 2005). Both LspA1 and LspA2 are delivered into host cells, where they undergo tyrosine phosphorylation by SFKs (Deng *et al*., 2008). The LspA proteins contain two 52-amino-acid segments repeated in the mid-region, each of which contains an EPIYA-related EPIYG motif (Fig. 1E). In addition, LspA1 has a 319-amino-acid repeat segment containing two EPIYA-related EPVYA motifs in its C-terminus, while LspA2 has three such motifs in its C-terminus (Ward *et al*., 1998; Deng *et al*., 2008). Whereas the pathophysiological role of LspA tyrosine phosphorylation remains to be elucidated, the observation that *H. ducreyi* inhibits phagocytosis by suppressing SFK activity raises the possibility that tyrosine-phosphorylated LspA activates Csk upon complex formation and thereby inactivates SFK as is the case of *H. pylori* CagA.

### Enteropathogenic *Escherichia coli* Tir

Enteropathogenic *E. coli* injects the Tir (translocated intimin receptor) protein via the TTSS into the cytoplasm of intestinal epithelial cells (Kenny *et al*., 1997). Once injected, Tir is localized to the plasma membrane with two membrane-spanning regions, making the N-terminal and C-terminal domains cytoplasmic and the central domain extracellular (Fig. 1F). The extracellular central domain specifically interacts with the *E. coli* outer membrane protein intimin. The Tir–intimin interaction triggers actin pedestal formation, which is important for the virulence of EPEC. The pedestal formation requires tyrosine phosphorylation of the translocated Tir protein at the EPIYA-related EHIYD motif by host kinases. Upon tyrosine phosphorylation, Tir interacts with the SH2 domain-containing adaptor protein Nck and the complex formation promotes actin polymerization (Kenny, 1999; Gruenheid *et al*., 2001; Campellone *et al*., 2002). Notably, enterohaemorrhagic *E. coli* (EHEC) also contains Tir, which induces actin pedestal formation. However, in contrast to EPEC Tir, EHEC Tir does not contain an EPIYA-related sequence motif and thus mechanisms of pedestal formation by the two related pathogenic *E. coli* strains may not be identical (Campellone *et al*., 2002).

## EPIYA motif – a pathogenic code exploited by bacteria?

A proteomic screening for mammalian protein interactors of bacterial effectors using 15-mer peptides derived from bacterial EPIYA motifs revealed that these peptides could promiscuously interact with an unusually large number of SH2 domain-containing mammalian proteins in a tyrosine phosphorylation-dependent manner (Selbach *et al*., 2009). Although protein interactions identified by the peptide-binding assay require formal validation using endogenous proteins, the observation led to the proposal by Selbach and Backert that the bacterial EPIYA motifs act as pathogenic ‘master keys’ that perturb multiple signalling pathways through promiscuous binding with SH2 domain-containing proteins (Backert *et al*., 2010). Notably, they reported that the sequence motif EPxYAxV (where x is any amino acid) is significantly underrepresented in mammalian proteomes. This may be due to negative selection of such a sequence motif because it could mediate inappropriate interaction with SH2-containing proteins that is harmful in mammalian cells. The host interactome with the bacterial EPIYA effectors is still under extensive research. Currently available list of the reported EPIYA effector–host protein interactions is provided in Table S1.

## Pragmin – a mammalian EPIYA-containing protein

Several bacteria have been shown to contain bacterial tyrosine kinase (BY-kinase) (Grangeasse *et al*., 2007). However, there is no evidence that bacterial EPIYA effectors are tyrosine-phosphorylated inside the bacteria. Also, the bacterial proteome does not possess SH2 domain-containing proteins, indicating that tyrosine phosphorylation-dependent interaction of EPIYA effector proteins never occurs in bacteria. Nevertheless, these bacterial effectors are capable of executing versatile interactions with mammalian SH2 domain-containing proteins. This observation raises the possibility that there is a mammalian EPIYA-containing protein(s), the function of which is exploited by bacterial effectors. If this is the case, it can be hypothesized that bacteria have independently invented EPIYA effectors so as to perturb/subvert the function of mammalian EPIYA-containing protein, which hampers successful bacterial infection. This idea prompted the identification of Pragmin, a mammalian cytoplasmic pseudokinase containing a single EPIYA motif that is phosphorylated by SFKs (Safari *et al*., 2011). Tyrosine-phosphorylated Pragmin binds to Csk and the interaction sequesters Csk to the cytoplasm, preventing its translocation to the plasma membrane, where it phosphorylates and inactivates SFKs. Consequently, Pragmin generates a positive feedback loop of SFK activation once it is tyrosine-phosphorylated (Safari *et al*., 2011). Of note, Csk appears to be frequently targeted by the bacterial EPIYA effectors (Tsutsumi *et al*., 2003; Selbach *et al*., 2009) and, indeed, *H. pylori* CagA competitively inhibits Pragmin–Csk interaction and recruits Csk to the plasma membrane, where it phosphorylates SFKs to inhibit their kinase activity. It is therefore possible that functional inhibition of SFK gives a substantial advantage to bacteria for successful infection, possibly by suppressing host innate immune responses as suggested in *H. ducreyi* infection (Mock *et al*., 2005). Importance of Csk in the function of EPIYA effectors is also supported by the observation that virtually all *H. pylori* CagA proteins contain at least one Csk-binding EPIYA segment (EPIYA-A or -B), although several CagA species do not possess an SHP2-binding EPIYA segment (EPIYA-C or -D). As SFKs are composed of nine different members, targeting the upstream regulator Csk may make it easier to systemically subvert SFKs than targeting each SFK family members one by one. In addition to CagA, EPIYA segments from several bacterial effectors such as BepD and BepE (Table S1) have been reported to bind to Csk. It is therefore possible that the Csk–Pragmin complex is a common target of several if not all of the bacterial EPIYA effectors to systemically perturb SFK functions. Notably, the sequence spanning the Pragmin EPIYA (EPIYAES) does not match EpxYAxV, the sequence that is profoundly depleted in mammalian proteomes. Pragmin EPIYA may therefore be permissive in mammalian cells because it does not exhibit promiscuous binding that perturbs mammalian cell function. This notion is supported by the observation that, as far as examined, Csk was the only SH2 domain-containing protein that bound to Pragmin in a tyrosine phosphorylation-dependent manner (Safari *et al*., 2011).

## Intrinsic disorder, a common feature of the bacterial EPIYA motifs

**Fig. 2:**
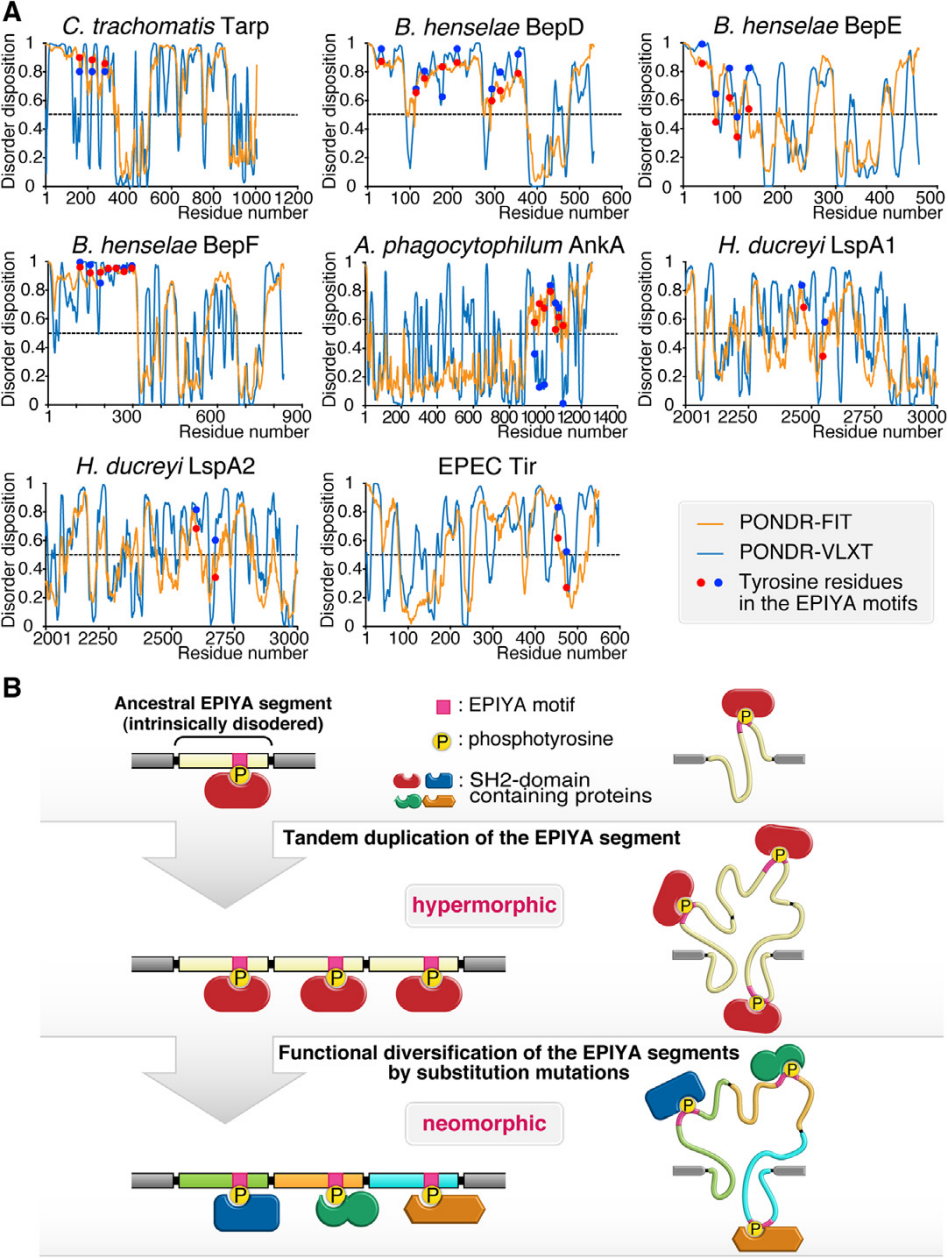
Intrinsic disorder, a driving force for EPIYA evolution? A. Structural disorder prediction for bacterial EPIYA effectors. Prediction for intrinsically disordered regions in bacterial effectors that possess the EPIYA-related motifs was performed using PONDR-FIT (http://www.disprot.org/pondr-fit) (orange) and PONDR-VLXT (http://www.pondr.com) (blue) predictors (Molecular Kinetics, http://www.pondr.com). Red and blue discs indicate the scores of tyrosine residues in the EPIYA-related motifs predicted by PONDR-FIT and PONDR-VLXT respectively. Access to PONDR® was provided by Molecular Kinetics (6201 La Pas Trail – Ste 160, Indianapolis, IN 46268, USA; 317-280-8737; E-mail: main@molecularkinetics.com). PONDR® is copyright©2004 by Molecular Kinetics, all rights reserved. B. A model for the evolution of bacterial EPIYA segments. An EPIYA segment, which contains an EPIYA tyrosine phosphorylation motif, can expand through tandem expansion via homologous recombination. Because of its intrinsically disordered nature, duplication of the EPIYA segment should not impose tertiary structural constraints on the protein. Increase in the number of the EPIYA segment potentiates the magnitude of EPIYA interaction with a target protein (hypermorphic allele). Introduction and accumulation of substitution mutations in each of duplicated EPIYA segments generate a new target-binding specificity (neomorphic allele) and thereby give rise to the functional diversification of the EPIYA segments.

The C-terminal region of *H. pylori* CagA, which contains the EPIYA motifs, lacks a stable tertiary structure (Nesic *et al*., 2010; Hayashi *et al*., 2012). The EPIYA-containing C-terminal region of EPEC Tir is also predicted to be natively unfolded (Race *et al*., 2007). The intrinsically disordered nature of the EPIYA motifs may enable versatile protein interaction because of its structural flexibility (Dyson and Wright, 2005; Dunker *et al*., 2008; Sigalov, 2010). Like many other disordered regions, different types of EPIYA segments in CagA may have evolved through repeated expansion of an ancestral EPIYA segment via homologous recombination and point mutations (Furuta *et al*., 2011). Prediction of protein disorder indicated that sequences spanning EPIYA motifs in other bacterial EPIYA effectors, especially Tarp, BepD, BepF and AnkA, are also intrinsically disordered (Fig. 2A). Tandem expansion of such a disordered EPIYA segment may maintain the unstructured nature of the region, while it quantitatively strengthens the ability of EPIYA effectors to interact with target proteins. Introduction and accumulation of substitution mutations into a duplicated EPIYA segment may then endow altered target-binding specificities, which could generate functional diversification of bacterial EPIYA effectors (Fig. 2B). Thus, the unstructured nature of the EPIYA segment may be a driving force that positively selects hypermorphic and/or neomorphic EPIYA effectors among various effector mutants non-specifically generated (see also *Conclusion*). It should also be noted that bacterial EPIYA effectors each have a unique EPIYA segments, which variably duplicate and align in individual EPIYA effector molecules. Such a structural variation within a given EPIYA effector species may underlie the differential pathogenic potential of individual effectors as demonstrated in *H. pylori* CagA (Higashi *et al*., 2002b; Naito *et al*., 2006) and *C. trachomatis* Tarp (Mehlitz *et al*., 2010).

Duplications of EPIYA segments may be achieved through homologous recombination of the nucleotide sequence encoding the segment (Furuta *et al*., 2011). In most cases, genomic duplication is neutral at the DNA level because it only extends the length of the duplex. In contrast, tandem duplication of peptides may create a serious problem if the segment on its own has a solid structure. A simple compilation of a solid structure may eventually insult the protein integrity and function. As a result, duplication of a gene segment which encoded a non-permissive protein structure had disappeared or will disappear during evolution. On the other hand, the lack of a stable tertiary structure (i.e. intrinsic disorder) in the duplication peptide unit renders the particular protein region functionally more active without causing structural constraints. We consider that this is a key strategy employed by pathogenic bacteria that enables generation and quick evolution of effector molecules to optimize their function inside host cells as a pathogenic scaffold/hub.

## Conclusion

It is highly possible that most if not all of the bacterial EPIYA effectors act as pathogenic scaffold/hub proteins upon delivery into mammalian host cells (Hatakeyama, 2003). This notion has been supported by the observation that CagA can genetically rescue loss-of-function mutation of Daughter of Sevenless (DOS), the *Drosophila* orthologue of mammalian Gab scaffold/adaptor protein (Botham *et al*., 2008). Mammalian scaffold proteins of Gab, IRS, Dok and Cas families are structurally made up of a solid N-terminal domain and an intrinsically disordered C-terminal extension (Simister and Feller, 2012). Recently, CagA was also found to be composed of an N-terminal structured region and a C-terminal disordered tail that contains EPIYA motifs (Hayashi *et al*., 2012). The conformational organization may be advantageous in the formation of a molecular platform that assembles multiple proteins. Hence, it would be interesting to determine whether bacterial EPIYA effectors share structural constitutions similar to those of mammalian scaffold proteins.

EPIYA motifs act as active sites of bacterial effectors that manipulate host cell signalling through tyrosine phosphorylation during host–pathogen interaction. A recent study using hidden Markov models showed that the EPIYA-containing proteins are significantly overrepresented in intracellular bacteria, extracellular bacteria with TTSS and TFSS or intracellular protozoan parasites (Xu *et al*., 2010). This may indicate that higher eukaryotes do not allow proteins with EPIYA motifs that can promiscuously interact with multiple SH2 domain-containing proteins. Alternatively, EPIYA-containing proteins might have been evolved convergently and/or distributed through horizontal transfer among bacteria because of the advantage in having such proteins during host–pathogen interaction. Systemic screening of EPIYA-containing proteins also predicted several potential bacterial EPIYA effectors (Xu *et al*., 2010). Without doubt, some of those effector candidates will turn out to be real EPIYA effectors in the near future. Identification of bacterial EPIYA effectors also points to the importance for development of compounds that specifically neutralize the bacterial EPIYA function as a new therapeutic strategy against bacterial infection.

## Supporting information

**Table S1.** EPIYA-mediated interaction of bacterial effectors with host proteins.

## References

[b1] Backert S, Selbach M (2005). Tyrosine-phosphorylated bacterial effector proteins: the enemies within. Trends Microbiol.

[b2] Backert S, Tegtmeyer N, Selbach M (2010). The versatility of *Helicobacter pylori* CagA effector protein functions: the master key hypothesis. Helicobacter.

[b3] Basso D, Zambon CF, Letley DP, Stranges A, Marchet A, Rhead JL (2008). Clinical relevance of *Helicobacter pylori cagA* and *vacA* gene polymorphisms. Gastroenterology.

[b4] Botham CM, Wandler AM, Guillemin K (2008). A transgenic *Drosophila* model demonstrates that the *Helicobacter pylori* CagA protein functions as a eukaryotic Gab adaptor. PLoS Pathog.

[b5] Campellone KG, Giese A, Tipper DJ, Leong JM (2002). A tyrosine-phosphorylated 12-amino-acid sequence of enteropathogenic *Escherichia coli* Tir binds the host adaptor protein Nck and is required for Nck localization to actin pedestals. Mol Microbiol.

[b7] Clifton DR, Fields KA, Grieshaber SS, Dooley CA, Fischer ER, Mead DJ (2004). A chlamydial type III translocated protein is tyrosine-phosphorylated at the site of entry and associated with recruitment of actin. Proc Natl Acad Sci USA.

[b6] Clifton DR, Dooley CA, Grieshaber SS, Carabeo RA, Fields KA, Hackstadt T (2005). Tyrosine phosphorylation of the chlamydial effector protein Tarp is species specific and not required for recruitment of actin. Infect Immun.

[b8] Covacci A, Rappuoli R (2000). Tyrosine-phosphorylated bacterial proteins: Trojan horses for the host cell. J Exp Med.

[b9] Deng K, Mock JR, Greenberg S, van Oers NSC, Hansen EJ (2008). *Haemophilus ducreyi* LspA proteins are tyrosine phosphorylated by macrophage-encoded protein tyrosine kinases. Infect Immun.

[b10] Dunker AK, Silman I, Uversky VN, Sussman JL (2008). Function and structure of inherently disordered proteins. Curr Opin Struct Biol.

[b11] Dyson HJ, Wright PE (2005). Intrinsically unstructured proteins and their functions. Nat Rev Mol Cell Biol.

[b12] Furuta Y, Yahara K, Hatakeyama M, Kobayashi I (2011). Evolution of *cagA* oncogene of *Helicobacter pylori* through recombination. PLoS ONE.

[b13] Grangeasse C, Cozzone A, Deutscher J, Mijakovic I (2007). Tyrosine phosphorylation: an emerging regulatory device of bacterial physiology. Trends Biochem Sci.

[b14] Gruenheid S, DeVinney R, Bladt F, Goosney D, Gelkop S, Gish GD (2001). Enteropathogenic *E. coli* Tir binds Nck to initiate actin pedestal formation in host cells. Nat Cell Biol.

[b15] Hatakeyama M (2003). *Helicobacter pylori* CagA – a potential bacterial oncoprotein that functionally mimics the mammalian Gab family of adaptor proteins. Microbes Infect.

[b16] Hatakeyama M (2004). Oncogenic mechanisms of *Helicobacter pylori* CagA protein. Nat Rev Cancer.

[b17] Hayashi T, Senda M, Morohashi H, Higashi H, Horio M, Kashiba Y (2012). Tertiary structure-function analysis reveals the pathogenic signaling potentiation mechanism of *Helicobacter pylori* oncogenic effector CagA. Cell Host Microbe.

[b19] Higashi H, Tsutsumi R, Muto S, Sugiyama T, Azuma T, Asaka M, Hatakeyama M (2002a). SHP-2 tyrosine phosphatase as an intracellular target of *Helicobacter pylori* CagA protein. Science.

[b18] Higashi H, Tsutsumi R, Fujita A, Yamazaki S, Asaka M, Azuma T, Hatakeyama M (2002b). Biological activity of the *Helicobacter pylori* virulence factor CagA is determined by variation in the tyrosine phosphorylation sites. Proc Natl Acad Sci USA.

[b20] Ijdo JW, Carlson AC, Kennedy EL (2007). *Anaplasma phagocytophilum* AnkA is tyrosine-phosphorylated at EPIYA motifs and recruits SHP-1 during early infection. Cell Microbiol.

[b21] Kenny B (1999). Phosphorylation of tyrosine 474 of the enteropathogenic *Escherichia coli* (EPEC) Tir receptor molecules is essential for actin nucleating activity and is preceded by additional host modifications. Mol Microbiol.

[b22] Kenny B, DeVinney R, Stein M, Reinscheid DJ, Frey EA, Finlay BB (1997). Enteropathogenic *E. coli* (EPEC) transfers its receptor for intimate adherence into mammalian cells. Cell.

[b23] Lane BJ, Mutchler C, Al Khodor S, Grieshaber SS, Carabeo RA (2008). Chlamydial entry involves TARP binding of guanine nucleotide exchange factors. PLoS Pathog.

[b24] Lin M, den Dulk-Ras A, Hooykaas PJJ, Rikihisa Y (2007). *Anaplasma phagocytophilum* AnkA secreted by type IV secretion system is tyrosine phosphorylated by Abl-1 to facilitate infection. Cell Microbiol.

[b25] Lutter EI, Bonner C, Holland MJ, Suchland RJ, Stamm WE, Jewett TJ (2010). Phylogenetic analysis of *Chlamydia trachomatis* Tarp and correlation with clinical phenotype. Infect Immun.

[b26] Mehlitz A, Banhart S, Hess S, Selbach M, Meyer TF (2008). Complex kinase requirements for *Chlamydia trachomatis* Tarp phosphorylation. FEMS Microbiol Lett.

[b27] Mehlitz A, Bänhart S, Maurer AP, Kaushansky A, Gordus AG, Zielecki J (2010). Tarp regulates early *Chlamydia*-induced host cell survival through interactions with the human adaptor protein SHC1. J Cell Biol.

[b29] Miura M, Ohnishi N, Tanaka S, Yanagiya K, Hatakeyama M (2009). Differential oncogenic potential of geographically distinct *Helicobacter pylori* CagA isoforms in mice. Int J Cancer.

[b30] Mock JR, Vakevainen M, Deng K, Latimer JL, Young JA, van Oers NS (2005). *Haemophilus ducreyi* targets Src family protein tyrosine kinases to inhibit phagocytic signaling. Infect Immun.

[b31] Naito M, Yamazaki T, Tsutsumi R, Higashi H, Onoe K, Yamazaki S (2006). Influence of EPIYA-repeat polymorphism on the phosphorylation-dependent biological activity of *Helicobacter pylori* CagA. Gastroenterology.

[b32] Nesic D, Miller MC, Quinkert ZT, Stein M, Chait BT, Stebbins CE (2010). *Helicobacter pylori* CagA inhibits PAR1-MARK family kinases by mimicking host substrates. Nat Struct Mol Biol.

[b33] Ohnishi N, Yuasa H, Tanaka S, Sawa H, Miura M, Matsui A (2008). Transgenic expression of *Helicobacter pylori* CagA induces gastrointestinal and hematopoietic neoplasms in mouse. Proc Natl Acad Sci USA.

[b34] Poppe M, Feller SM, Römer G, Wessler S (2007). Phosphorylation of *Helicobacter pylori* CagA by c-Abl leads to cell motility. Oncogene.

[b35] Race PR, Solovyova AS, Banfield MJ (2007). Conformation of the EPEC Tir protein in solution: investigating the impact of serine phosphorylation at positions 434/463. Biophys J.

[b36] Rosenshine I, Donnenberg MS, Kaper JB, Finlay BB (1992). Signal transduction between enteropathogenic *Escherichia coli* (EPEC) and epithelial cells: EPEC induces tyrosine phosphorylation of host cell proteins to initiate cytoskeletal rearrangement and bacterial uptake. EMBO J.

[b37] Safari F, Murata-Kamiya N, Saito Y, Hatakeyama M (2011). Mammalian Pragmin regulates Src family kinases via the Glu-Pro-Ile-Tyr-Ala (EPIYA) motif that is exploited by bacterial effectors. Proc Natl Acad Sci USA.

[b38] Schulein R, Guye P, Rhomberg TA, Schmid MC, Schröder G, Vergunst AC (2005). A bipartite signal mediates the transfer of type IV secretion substrates of *Bartonella henselae* into human cells. Proc Natl Acad Sci USA.

[b39] Selbach M, Paul FE, Brandt S, Guye P, Daumke O, Backert S (2009). Host cell interactome of tyrosine-phosphorylated bacterial proteins. Cell Host Microbe.

[b40] Sigalov AB (2010). Protein intrinsic disorder and oligomericity in cell signaling. Mol Biosyst.

[b41] Simister PC, Feller SM (2012). Order and disorder in large multi-site docking proteins of the Gab family – implications for signalling complex formation and inhibitor design strategies. Mol Biosyst.

[b42] Suzuki M, Mimuro H, Suzuki T, Park M, Yamamoto T, Sasakawa C (2005). Interaction of CagA with Crk plays an important role in *Helicobacter pylori*-induced loss of gastric epithelial cell adhesion. J Exp Med.

[b43] Tammer I, Brandt S, Hartig R, König W, Backert S (2007). Activation of Abl by *Helicobacter pylori*: a novel kinase for CagA and crucial mediator of host cell scattering. Gastroenterology.

[b44] Tsutsumi R, Higashi H, Higuchi M, Okada M, Hatakeyama M (2003). Attenuation of *Helicobacter pylori* CagA x SHP-2 signaling by interaction between CagA and C-terminal Src Kinase. J Biol Chem.

[b45] Vakevainen M, Greenberg S, Hansen EJ (2003). Inhibition of phagocytosis by *Haemophilus ducreyi* requires expression of the LspA1 and LspA2 proteins. Infect Immun.

[b48] Ward CK, Lumbley SR, Latimer JL, Cope LD, Hansen EJ (1998). *Haemophilus ducreyi* secretes a filamentous hemagglutinin-like protein. J Bacteriol.

[b9001] Ward CK, Latimer JL, Nika J, Vakevainen M, Mock JR, Deng K (2003). Mutations in the *lspA1* and *lspA2* genes of *Haemophilus ducreyi* affect the virulence of this pathogen in an animal model system. Infect Immun.

[b46] Xu S, Zhang C, Gao J, Xu D (2010). Effector prediction in host-pathogen interaction based on a Markov model of a ubiquitous EPIYA motif. BMC Genomics.

[b47] Yong EC, Chi EY, Kuo CC (1987). Differential antimicrobial activity of human mononuclear phagocytes against the human biovars of *Chlamydia trachomatis*. J Immunol.

