# Supplementary material for: Bacterial EPIYA effectors – Where do they come from? What are they? Where are they going?
Source: Cell Microbiol. 2012 Nov 1;15(3):377–85. doi: 10.1111/cmi.12040 (PMC3593179; doi:10.1111/cmi.12040)
Supplement: Supplementary file 1 [file cmi0015-0377-SD1.zip › References to Supplementary Table 1.docx]

**References to Supplemental Table 1**

Campellone, K.G., Giese, A., Tipper, D.J., and Leong, J.M. (2002) A tyrosine-phosphorylated 12-amino-acid sequence of enteropathogenic *Escherichia coli* Tir binds the host adaptor protein Nck and is required for Nck localization to actin pedestals. *Mol Microbiol* **43:** 1227-1241.

Gruenheid, S., DeVinney, R., Bladt, F., Goosney, D., Gelkop, S., Gish, G.D., Pawson, T., and Finlay, B.B. (2001) Enteropathogenic *E. coli* Tir binds Nck to initiate actin pedestal formation in host cells. *Nat Cell Biol* **3:** 856-859.

Higashi, H., Tsutsumi, R., Muto, S., Sugiyama, T., Azuma, T., Asaka, M., and Hatakeyama, M. (2002) SHP-2 tyrosine phosphatase as an intracellular target of *Helicobacter pylori* CagA protein. *Science* **295:** 683-686.

Ijdo, J.W., Carlson, A.C., and Kennedy, E.L. (2007) *Anaplasma phagocytophilum* AnkA is tyrosine-phosphorylated at EPIYA motifs and recruits SHP-1 during early infection. *Cell Microbiol* **9:** 1284-1296.

Lane, B.J., Mutchler, C., Al Khodor, S., Grieshaber, S.S., and Carabeo, R.A. (2008) Chlamydial entry involves TARP binding of guanine nucleotide exchange factors. *PLoS Pathog* **4:** e1000014.

Mehlitz, A., Bänhart, S., Maurer A.P., Kaushansky, A., Gordus, A.G., Zielecki, J., Macbeath, G., and Meyer, T.F. (2010) Tarp regulates early *Chlamydia*-induced host cell survival through interactions with the human adaptor protein SHC1. *J Cell Biol* **190:** 143-157.

Mimuro, H., Suzuki, T., Tanaka, J., Asahi, M., Haas, R., and Sasakawa, C. (2002) Grb2 is a key mediator of *Helicobacter pylori* CagA protein activities.

Safari, F., Murata-Kamiya, N., Saito, Y., and Hatakeyama, M. (2011) Mammalian Pragmin regulates Src family kinases via the Glu-Pro-Ile-Tyr-Ala (EPIYA) motif that is exploited by bacterial effectors. *Proc Natl Acad Sci USA* **108:** 14938-14943.

Selbach, M., Paul, F.E., Brandt, S., Guye, P., Daumke, O., Backert, S., Dehio, C., and Mann, M. (2009) Host cell interactome of tyrosine-phosphorylated bacterial proteins. *Cell Host Microbe* **5:** 397-403.

Suzuki, M., Mimuro, H., Suzuki, T., Park, M., Yamamoto, T., and Sasakawa, C. (2005) Interaction of CagA with Crk plays an important role in *Helicobacter pylori*-induced loss of gastric epithelial cell adhesion. *J Exp Med* **202:** 1235-1247.

Tsutsumi, R., Higashi, H., Higuchi, M., Okada, M., and Hatakeyama, M. (2003) Attenuation of *Helicobacter pylori* CagA x SHP-2 signaling by interaction between CagA and C-terminal Src Kinase. *J Biol Chem* **278:** 3664-3670.

Tsutsumi, R., Takahashi, A., Azuma, T., Higashi, H., and Hatakeyama, M. (2006) Focal adhesion kinase is a substrate and downstream effector of SHP-2 complexed with *Helicobacter pylori* CagA. *Mol Cell Biol* **26:** 61-76.
