# Supplementary material for: Bacterial EPIYA effectors – Where do they come from? What are they? Where are they going?
Source: Cell Microbiol. 2012 Nov 1;15(3):377–85. doi: 10.1111/cmi.12040 (PMC3593179; doi:10.1111/cmi.12040)
Supplement: Supplementary file 1 [file cmi0015-0377-SD1.zip › Suppl_Table_1.docx]

Supplemental Table 1. EPIYA-mediated interaction of bacterial effectors with host proteins

| Organism | EPIYA effector | EPIYA-like motif | pY site | Interacting partner | Pathogenic function | Experimental method | Reference |
| --- | --- | --- | --- | --- | --- | --- | --- |
| *H. pylori*  (26695) | CagA | EPIYA  (A site) | 899 | Csk | inhibition of SFK activity  reduces CagA toxicity | co-IP (T)  SILAC/MS | Tsutsumi et al. 2003 Selbach et al. 2009 |
|  |  |  |  | SHP1 | unknown | SILAC/MS  co-IP (I) | Selbach et al. 2009 |
|  |  | EPIYT  (B site) | 918 | Csk | inhibition of SFK activity  reduces CagA toxicity | co-IP (T)  SILAC/MS | Tsutsumi et al. 2003 Selbach et al. 2009 |
|  |  |  |  | Grb2 | cell elongation/scattering  activation of Ras-MAPK signaling | SILAC/MS | Selbach et al. 2009  Mimuro et al. 2002 |
|  |  |  |  | SHP1 | unknown | SILAC/MS  co-IP (I) | Selbach et al. 2009 |
|  |  |  |  | SHP2 | cell elongation/scattering  activation of Erk-MAPK signaling  Dephosphorylation of FAK | co-IP (T)  SILAC/MS | Higashi et al. 2002  Tsutsumi et al. 2006  Selbach et al. 2009 |
|  |  |  |  | PI3K (p85) | unknown | SILAC/MS  co-IP (I) | Selbach et al. 2009 |
|  |  | EPIYA  (C site) | 972 | Grb2 | cell elongation/scattering  activation of Ras-MAPK signaling | SILAC/MS | Selbach et al. 2009  Mimuro et al. 2002 |
|  |  |  |  | Grb7 | unknown | SILAC/MS  co-IP(I) | Selbach et al. 2009 |
|  |  |  |  | RasGAP | unknown | SILAC/MS | Selbach et al. 2009 |
|  |  |  |  | SHP1 | unknown | SILAC/MS  co-IP (I) | Selbach et al. 2009 |
|  |  |  |  | SHP2 | cell elongation/scattering  activation of Erk-MAPK signaling  Dephosphorylation of FAK | co-IP (T)  SILAC/MS | Higashi et al. 2002  Tsutsumi et al. 2006  Selbach et al. 2009 |
|  |  |  | ? | Crk | cell elongation/scattering  disrupts adherens junctions  activation of MAPK signaling | co-IP (I) | Suzuki et al. 2005 |
| *C.trachom-atis*  (serovar L2) | Tarp | ENIYE | 189/  291/  341/ | Vav2 | unknown | oligopeptide pull-down  protein microarray | Lane et al. 2008  Mehlitz et al.2010 |
|  |  |  |  | Shc1 | anti-apoptotic  MEK, ERK activation | protein microarray  co-IP (I) | Mehlitz et al. 2010 |
|  |  |  |  | Nck2 | unknown | protein microarray  pull-down | Mehlitz et al. 2010 |
|  |  |  |  | PI3K | unknown | protein microarray | Melhitz et al. 2010 |
|  |  |  |  | RasGAP | unknown | protein microarray | Melhitz et al. 2010 |
|  |  |  |  | Syk | unknown | protein microarray | Melhitz et al. 2010 |
|  |  |  |  | Hck | unknown | protein microarray | Melhitz et al. 2010 |
|  |  |  |  | Fgr | unknown | protein microarray | Melhitz et al. 2010 |
|  |  | ENIYENIYE | 136/  238/  390/  140/  238/  390 | CrkL | unknown | protein microarray | Melhitz et al. 2010 |
|  |  |  |  | Plcg2 | unknown | protein microarray | Melhitz et al. 2010 |
|  |  |  |  | Shb | unknown | protein microarray | Melhitz et al. 2010 |
|  |  |  |  | Grb2 | unknown | protein microarray | Melhitz et al. 2010 |
|  |  |  |  | RasGAP | unknown | protein microarray | Melhitz et al. 2010 |
|  |  |  |  | Abl1 | unknown | protein microarray | Melhitz et al. 2010 |
|  |  |  |  | Abl2 | unknown | protein microarray | Melhitz et al. 2010 |
|  |  |  |  | Shc1 | anti-apoptotic  MEK, ERK activation | protein microarray  pull-down | Melhitz et al. 2010 |
|  |  |  |  | Shc3 | unknown | protein microarray | Melhitz et al. 2010 |
|  |  |  |  | Nck2 | unknown | protein microarray | Melhitz et al. 2010 |
|  |  |  |  | Pik3 | unknown | protein microarray | Melhitz et al. 2010 |
|  |  |  |  | Vav2 | unknown | protein microarray | Melhitz et al. 2010 |
|  |  |  |  | Syk | unknown | protein microarray | Melhitz et al. 2010 |
|  |  |  |  | Fgr | unknown | protein microarray | Melhitz et al. 2010 |
|  |  |  |  | Hck | unknown | protein microarray | Melhitz et al. 2010 |
|  |  |  |  | Yes1 | unknown | protein microarray | Melhitz et al. 2010 |
|  |  |  |  | Sh3bp2 | unknown | protein microarray | Melhitz et al. 2010 |
|  |  |  |  | Vav1 | unknown | protein microarray | Melhitz et al. 2010 |
|  |  |  |  | Crk | unknown | protein microarray | Melhitz et al. 2010 |
|  |  |  |  | Lck | unknown | protein microarray | Melhitz et al. 2010 |
| *C.trachom-atis* (serovar D) | Tarp | ENIYE | 171/  221/  271 | Cbl-B | unknown | SILAC/MS | Selbach et al. 2009 |
|  |  |  |  | Cbl-C | unknown | SILAC/MS | Selbach et al. 2009 |
|  |  |  |  | Grb2 | unknown | SILAC/MS | Selbach et al. 2009 |
|  |  |  |  | Grb7 | unknown | SILAC/MS | Selbach et al. 2009 |
|  |  |  |  | PI3K | unknown | SILAC/MS | Selbach et al. 2009 |
|  |  |  |  | Shc1 | anti-apoptotic  MEK, ERK activation | protein microarray  pull-down | Mehlitz et al. 2010 |
|  |  |  |  | Shc3 | unknown | protein microarray | Mehlitz et al. 2010 |
|  |  |  |  | Abl1 | unknown | protein microarray | Mehlitz et al. 2010 |
|  |  |  |  | CrkL | unknown | protein microarray | Mehlitz et al. 2010 |
|  |  |  |  | RasGAP | unknown | protein microarray | Mehlitz et al. 2010 |
| *B.henselae*  (ATCC49882) | BepD | EPLYA | 32 | SHP2 | unknown | SILAC/MS | Selbach et al. 2009 |
|  |  |  | 211 | Csk | unknown | SILAC/MS | Selbach et al. 2009 |
|  |  |  |  | Grb2 | unknown | SILAC/MS | Selbach et al. 2009 |
|  |  |  |  | Grb7 | unknown | SILAC/MS | Selbach et al. 2009 |
|  |  |  |  | SHP1 | unknown | SILAC/MS | Selbach et al. 2009 |
|  |  |  |  | SHP2 | unknown | SILAC/MS | Selbach et al. 2009 |
|  | BepE | EPLYA | 37 | Csk | unknown | SILAC/MS  co-IP (T) | Selbach et al. 2009 |
|  |  | EVVYA | 64 | Grb2 | unknown | SILAC/MS | Selbach et al. 2009 |
|  |  |  |  | Grb7 | unknown | SILAC/MS | Selbach et al. 2009 |
|  |  |  |  | SHP1 | unknown | SILAC/MS | Selbach et al. 2009 |
|  |  |  |  | SHP2 | unknown | SILAC/MS  co-IP (T) | Selbach et al. 2009 |
|  | BepF | TPLYA | 149 | CrkL | unknown | SILAC/MS | Selbach et al. 2009 |
|  |  |  |  | Grb2 | unknown | SILAC/MS | Selbach et al. 2009 |
|  |  |  |  | RasGAP | unknown | SILAC/MS | Selbach et al. 2009 |
|  |  | EPLYA | 213 | Crk | unknown | SILAC/MS | Selbach et al. 2009 |
| *A. phagocytop-hilum*  (NCH-1) | AnkA | ESIYE (A site)  EDLYA (B site)  ESIYA (C site)  EPIYA (D site) | ? | SHP1 | unknown | co-IP (I) | IJdo et al. 2007 |
| *EPEC*  (E2348/69) | Tir | VNPYA | 454 | Cbl-C | unknown | SILAC/MS | Selbach et al. 2009 |
|  |  |  |  | PI3K (p85) | unknown | SILAC/MS  co-IP (I) | Selbach et al. 2009 |
|  |  |  |  | SHP2 | unknown | SILAC/MS | Selbach et al. 2009 |
|  |  | EHIYD | 474 | Nck1 | actin polymerization | pull-down  SILAC/MS | Gruenheld et al. 2001  Campellone et al. 2002 Selbach et al. 2009 |
|  |  |  |  | Nck2 | actin polymerization | SILAC/MS | Selbach et al. 2009  Gruenheld et al. 2001 |
|  |  |  |  | RasGAP | unknown | SILAC/MS | Selbach et al. 2009 |
|  |  |  |  | Src | unknown | SILAC/MS | Selbach et al. 2009 |
| *H. sapiens* | Pragmin | EPIYA | 391 | Csk | inhibition of SFK activity | co-IP (endogenous) | Safari et al. 2011 |

(I): Infection, (T): Transfection, (SILAC): stable isotope labeling using amino acids in cell culture, (MS): mass-spectrometry, (co-IP): co-immunoprecipitation, (pY): tyrosine phosphorylation.
